# Evidence of a hormonal reshuffle in the cecal metabolome fingerprint of a strain of rats resistant to decompression sickness

**DOI:** 10.1038/s41598-021-87952-y

**Published:** 2021-04-15

**Authors:** Nicolas Vallee, Emmanuel Dugrenot, Anne-Virginie Desruelle, Catherine Tardivel, Jean-Charles Martin, Anthony Guernec, Alain Boussuges, Sarah Rives, Jean-Jacques Risso, François Guerrero

**Affiliations:** 1 Institut de Recherche Biomédicale des Armées, Equipe de Recherche Subaquatique Opérationnelle, 83800 Toulon Cedex 9, France; 2 Univ Brest, ORPHY, IBSAM, 29200 Brest, France; 3 C2VN, INRAE, INSERM, BIOMET, Aix Marseille Univ, Marseille, France

**Keywords:** Physiology, Metabolomics

## Abstract

On one side, decompression sickness (DCS) with neurological disorders lead to a reshuffle of the fecal metabolome from rat caecum. On the other side, there is high inter-individual variability in terms of occurrence of DCS. One could wonder whether the fecal metabolome could be linked to the DCS-susceptibility. We decided to study male and female rats selected for their resistance to decompression sickness, and we hypothesize a strong impregnation concerning the fecal metabolome. The aim is to verify whether the rats resistant to the accident have a fecal metabolomic signature different from the stem generations sensitive to DCS. 39 DCS-resistant animals (21 females and 18 males), aged 14 weeks, were compared to 18 age-matched standard Wistar rats (10 females and 8 males), i.e., the same as those we used for the founding stock. Conventional and ChemRICH approaches helped the metabolomic interpretation of the 226 chemical compounds analyzed in the cecal content. Statistical analysis shows a panel of 81 compounds whose expression had changed following the selection of rats based on their resistance to DCS. 63 compounds are sex related. 39 are in common. This study shows the spectral fingerprint of the fecal metabolome from the caecum of a strain of rats resistant to decompression sickness. This study also confirms a difference linked to sex in the metabolome of non-selected rats, which disappear with selective breeding. Results suggest hormonal and energetic reshuffle, including steroids sugars or antibiotic compounds, whether in the host or in the microbial community.

## Introduction

In scuba diving or when working under pressure, the inhaled gases dissolve in the organism tissues as pressure increases. These gases may then give rise to bubbles during the desaturation phase. Symptoms of decompression sickness (DCS) may appear following an excessive quantities of bubbles in the organism^1^. It is recognized that the amount of venous bubbles is a positively correlated to the risk of DCS^2,3^. However, for a same dive profile, the intra- and inter-individual variability is high in terms of bubble quantity and occurrence of DCS.

Among the multiplicity of identified factors that can lead to DCS, such as age^4^, weight^5^ or the presence of a permeable foramen ovale^6^, it also seems that inherited characteristics, such as sex^7,8^, can be much influential^9^. In fact, the heritable component of DCS was able to be highlighted through the selection and then the breeding of rats resistant to DCS^9,10^.

Interestingly, the composition of the gut microbiota is strongly impacted by the genetic background of the animal strain, perhaps even more than by maternal inoculation or exposure to a different exogenous microbiota^11^. While many studies now emphasized the similarities of the gut microbiota between rats and humans^12–14^, It has recently been demonstrated that the digestive tract, through the contribution of the intestinal microbiota, could also have an influence on the occurrence of DCS^15–20^. The present study is particularly devoted to the selection of a fecal metabolome operated upon generation of rat strains resistant to DCS. Metabolomics analysis was used to indirectly assesses, by measuring the compounds present, the activity of the microbiota of the digestive system with its host.

This study is an extension of previous work^9,10^ where rats without DCS were selected and bred to create a new generation, then subjected to the same hyperbaric protocol. This procedure was repeated and the proportion of DCS could be drastically reduced from 65 to 35% in the 3rd generation and 23% in the 6th generation^9,10^. Moreover, sex-dependent differences were observed in both the gain of resistance to DCS throughout generations and the physiological modifications associated with resistance to DCS.

Here, we studied male and female rats selected for their resistance to decompression sickness, and we hypothesize a strong impregnation concerning the fecal metabolome from caecum. The aim is to verify whether the rats resistant to the accident have a fecal metabolomic signature different from the stem generations sensitive to DCS.

## Methods

### Animals and ethical statement

All procedures involving experimental animals follow the 3Rs and complied with European Union rules (Directive 2010/63/EU), French law (Decree 2013/118) and the ARRIVE guideline. The Ethics Committee of the Université de Bretagne Occidentale approved this study (Approval No.; APAFIS#10395-2017061909495511). The founder population was composed from 6-wk old Wistar rats (Janvier SAS, Geneste, France). They were housed in an accredited animal care facility, under controlled temperature (22 ± 1 °C) and lighting (12 h of light per day, 6:00 am–6:00 pm). They were fed standard rat kibble and water were provided ad libitum.

### Animals

39 DCS-resistant (Res) animals (21 females and 18 males), aged 14 weeks, bred at the university animal house were used in this study. They were compared to 18 age-matched standard (Std) Wistar rats (10 females and 8 males), i.e., the same as those we used for the founding stock, obtained from the same breeder (Janvier Labs, St Genêts, France). Because our aim was to study the differences in the fecal metabolome associated with resistance to DCS independently of possible persistent modifications induced by diving itself^21–23^ none of these individuals were previously exposed to hyperbaric treatment. The standard rats were acclimated with the facility for at least 2 weeks. All animals were housed three per cage under controlled temperature (21 ± 1 °C) and lighting (12 h of light per day, 0600–1800) at the university animal housing facility until the day of the day of cecal content harvesting. They were fed standard rat chow and water ad libitum.

### Fecal metabolome

Animals were anaesthetized first by administration of gaseous isoflurane (4.0% in 100% air flow at 2.0 l/min) through face mask for 5 min before intraperitoneal injection of ketamine (100 mg kg^−1^ body weight) and xylazine (10 mg kg^−1^ body weight). Anaesthesia level was determined by testing the lack of withdrawal reflexes in response to pinches of the distal hind limbs. The cecum was separated from the rest of the digestive tract after ligation. The cecum was weighed and then opened using a scalpel. Part of its contents was placed in a 1.5-ml Eppendorf tube and kept at − 80 °C until metabolomic analysis. Finally, animals were euthanized with a lethal dose of anesthetic.

This technique is similar to that previously used in our studies^23^. 100–150 mg of cecal content were homogenized in cooled methanol (3µL/mg feces) at  − 20 °C. Samples were vortexed for 1 min and incubated at  − 20 °C for 30 min. Samples were then centrifuged for 15 min (11,000 × g, 4 °C). The supernatant recovered from each sample was filtered through 10 KDa filter tubes by centrifuging for 45 min (11,000 × g, 4 °C). The extracts obtained were then dried using a stream of nitrogen and then frozen at  − 80 °C.

LCMS metabolomic analyses were performed essentially as described earlier^24^. All the dried polar extracts were first reconstituted with 150 µl acetonitrile/water (50:50; v:v). The samples were separated using high performance liquid chromatography (UPLC) ultimate 3000 (Thermo Scientific), coupled to a high-resolution Q-Exactive Plus quadrupole-orbitrap hybrid mass spectrometer (HRMS), equipped with electrospray ionization source (H-ESI II). The chromatographic separation was performed on a binary solvent system using a HILIC column (Merk,SeQuant ZIC-HILIC, 150 mm × 2.1 mm, 5 μm, 200 A) at 25 °C with a flow rate of 0.25 ml min^−1^. The injection volume was 5 μl. The mobile phase consisted of a combination of solvent A (100% water, 16 mM ammonium formate) and solvent B (100% acetonitrile 0.1% formic acid). The following gradient conditions were used: 0 to 2 min, isocratic 97% B;, 2 to 10 min, linear from 97 to 70% B; 10 to 15 min, linear 70 to 10% B; 15 to 17 min, isocratic 10% B; 17 to 18 min linear from 10 to 97% B; from 18 to 22 min isocratic 97% B. The separated molecules were analyzed in both positive and negative ionization modes in the same run (switch mode). The repeatability of the analysis was checked by analyzing interspaced (1 out of every 5 samples) quality control samples (QC).

Data processing and molecule identification: All the raw data generated by the LCMS were converted to mzXML by ProteoWizard (Version 2.0), and then processed by MZmine 2.26.

The data processing was performed in different steps. The first step was peak picking and required the application of the “CentWave” algorithm, the method of choice for processing centroided data acquired by HRMS. After extracting the mass to charge ratio (m/z), the retention time and the intensity for every ion, the peak picking step is then followed by isotope grouping, alignment and gap filling. The data were further processed to eliminate artifacts such as signals found in the blank, peaks without Gaussian shape or corresponding to noise. The analytical drift over the sample runs is then corrected by applying a linear correction as described by Van der Kloet et al.^25^. Then all features that represent over 30% of the deviation from the median by applying the relative standard deviation filtration were removed (a coefficient of variation less than 30% on QC samples peaks was applied to all samples). All these steps generally leave 10–20% of the initial features.

Metabolites annotation was then performed using the post-processed dataset. An in-house database referencing more than 800 metabolites with their chromatographic retention time acquired with a HILIC column, together with their exact mass and MSMS spectra obtained in positive and negative ionization modes, including their adducts and neutral losses. Matching the two datasets was performed using the “bank inhouse” tool of the W4M web application (https://workflow4metabolomics.usegalaxy.fr/) ^26^. Uncertainties were removed by comparing the MSMS spectra to that of our database or found in publicly available databases (HMDB, Mass Bank, MzCloud, Metlin). These led to Level 1 (MSMS, retention time, MS) or 2 identification (retention time, MS). Finally, 226 metabolites per rat were obtained.

### Statistical analyses

Statistical analyses were performed essentially as described in our previous study^23^. Most series of values fell between 0 and 1 and the distribution was positively skewed. Prior analysis, scale-contracting transformation was applied with log(X + 1). The difference was analyzed using 2-way ANOVA (type III SS) on clinical status and diet, comprising interactions, followed by post-hoc Tukey’s (HSD) and Benjamini-Hochberg’s (False Rate Discovery) tests. Principal component analysis (Pearson correlation coefficient), agglomerative hierarchical clustering (AHC) (dissimilarity; Euclidean distance; Ward’s method) helped by k-means clustering were used to design the heat map and volcano plot, from normalized data of the 226 features of the 57 diving rats. A random forest classifier (Sampling: random with replacement; method: random input) was also used in order to extract the most contributing compounds. The software was XlStat Biomed from Addinsoft. Maximum accepted alpha level was 5%.

## Results

### Metabolic analysis of feces

**Figure 1:**
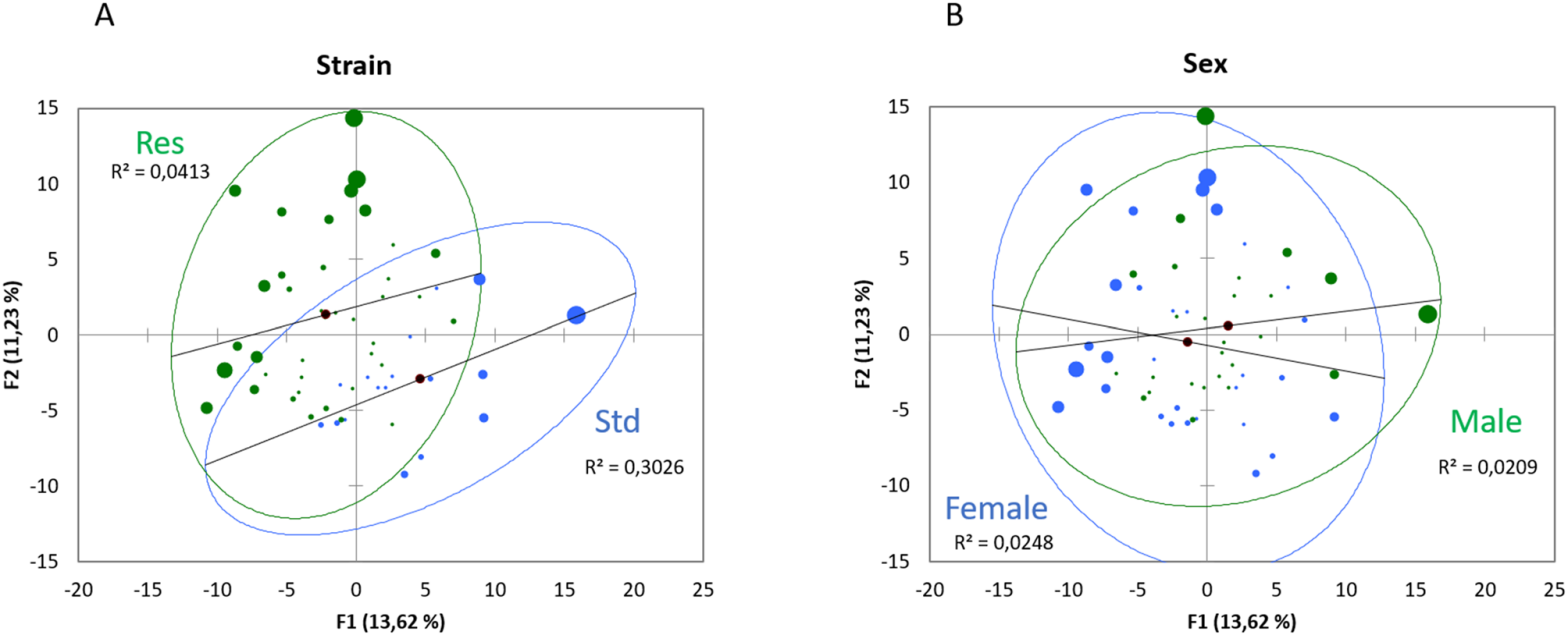
Principal component analysis (PCA) plot of fecal metabolome as function of (**A**) strain (Std and Res) or (**B**) sex.

226 metabolites were analyzed. Principal component analysis (PCA; Pearson test) (Fig. 1) of the fecal metabolome from rat caecum reveals two partially overlapped groups, associated with DCS-resistance, where the axes F1 and F2 explain 25% of the variability. A similar PCA to reveal a gender effect did not show any effect for this factor.

**Figure 2:**
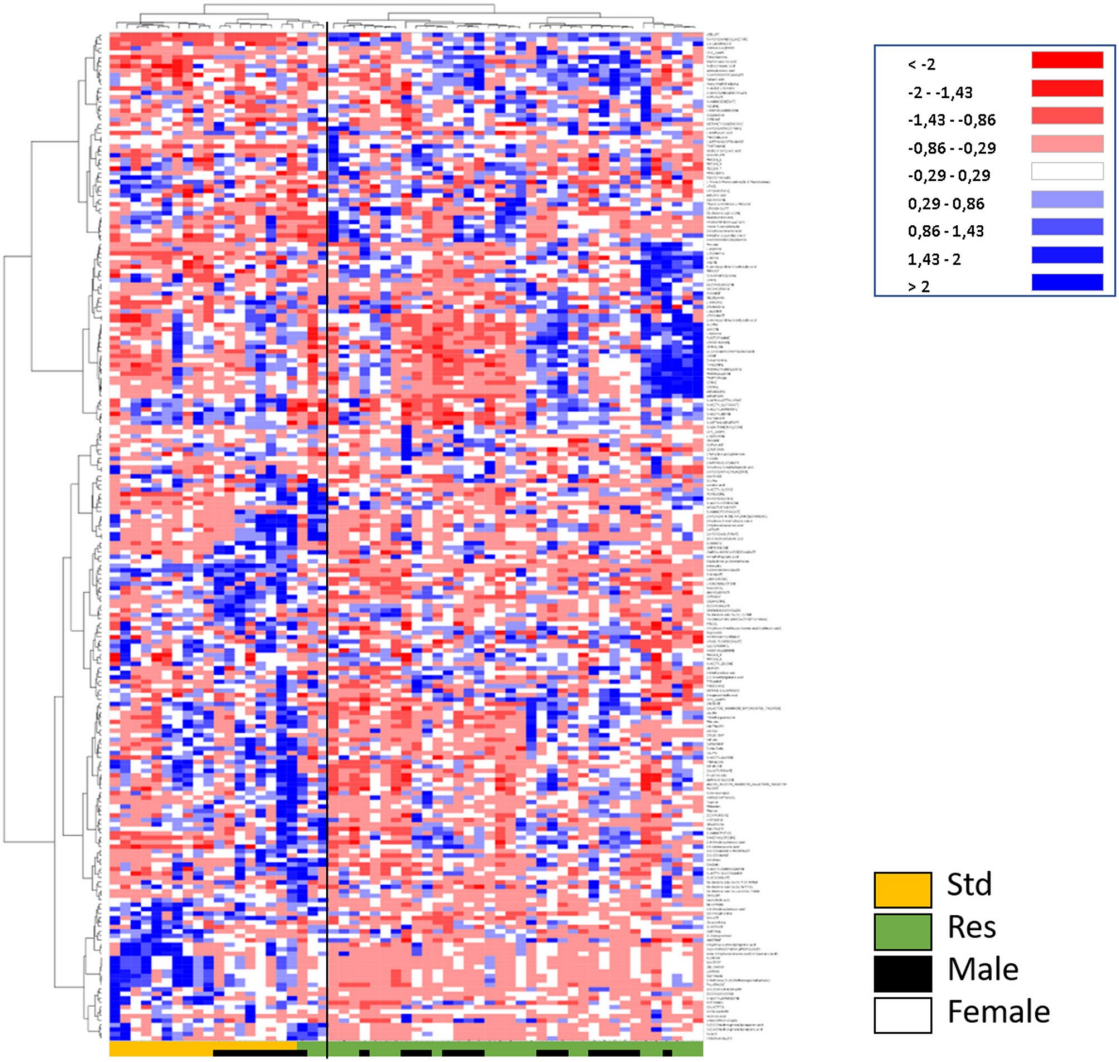
Heat Map, i.e. hierarchical clustering of fecal metabolomes from Std (orange) or Res (green) rats. Rats are on the ordinate where black squares note males. Fold-change for each metabolite (abscissa) is represented by a color. Intensity values are normalized, from red (Min:  − 2) to blue (Max: + 2).

An Ascending Hierarchical Classification (AHC) was carried out as part of the Heat Map established according to the intensity of the 226 compounds analyzed (Fig. 2). Following the evolution of the variances according to the number of classes, two inflection points are located in the 2nd and 4th class. The division into 2 classes explains 97% of the intragroup variability. According to this division, the first class (I) contains 18 Std and 3 Res rats, while the second class (II) contains only Res rats.

The division into 4 classes explains 74% of of the intragroup variability. Its first class Ia contains 10 Std rats with only females, and the second class (Ib) contains 8 male Std rats and 3 Res rats (including 1 female). The class IIa contains 17 Res rats with 7 females, and the last one (IIb) contains 19 Res with 11 females.

The level of metabolite expression is marked according to the strain, with also a pronounced dichotomy between males and females in standard rats. This difference between males and females is no longer observed in Res.

The AHC of the Heat Map allows to visualize on the abscissa a separation of the metabolites between the Std rats on the left (orange) and the Res rats on the right (green). In this same left part of the map in Std (orange), an over-expression of fecal compounds in female rats is visible in blue on the lower part. Offset, just above, an over-expression of the compounds (in blue) is visible in male Std rats. Differences between males and females are no longer so obvious in Res (lower part of the heat map, notably).

More generally, dichotomies appear at the ends of the abscissa between the Std and the Res, as well as between the compounds presented at the ends of the ordinate. Std females are clearly separated from Std males (black, orange), but this is no more true in Res animals (green).

**Figure 3:**
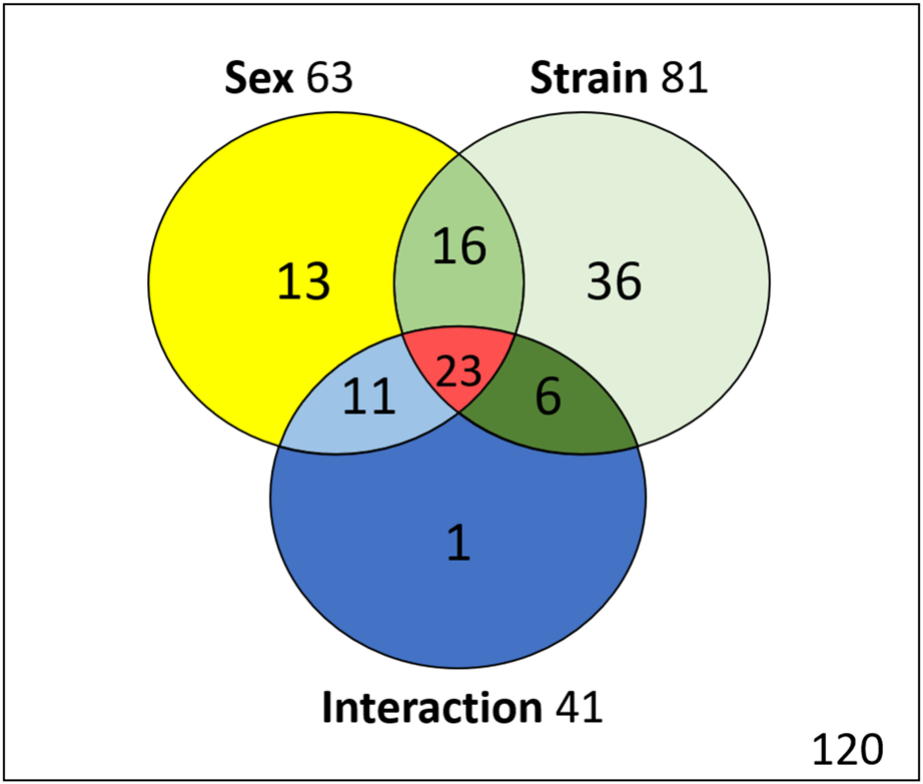
Venn diagram with metabolites influenced by sex or strain, and their interactions.

The ANOVA (Type III SS with post-hoc Tukey (HSD) and Benjamini-Hochberg (FRD)) with two factors (Sex and Strain) has been performed for the 226 compounds in the 57 rats. The Venn diagram (Fig. 3 + Supplementary data 1 for the list of metabolites) allows to resume the effects linked to gender or strain, as well as their interactions. Out of the 226 compounds analyzed, 63 were significantly influenced by the sex and 81 differed with resistance. 41 interactions are noted including 23 synergies.

### Metabolomic analysis on gender

**Figure 4:**
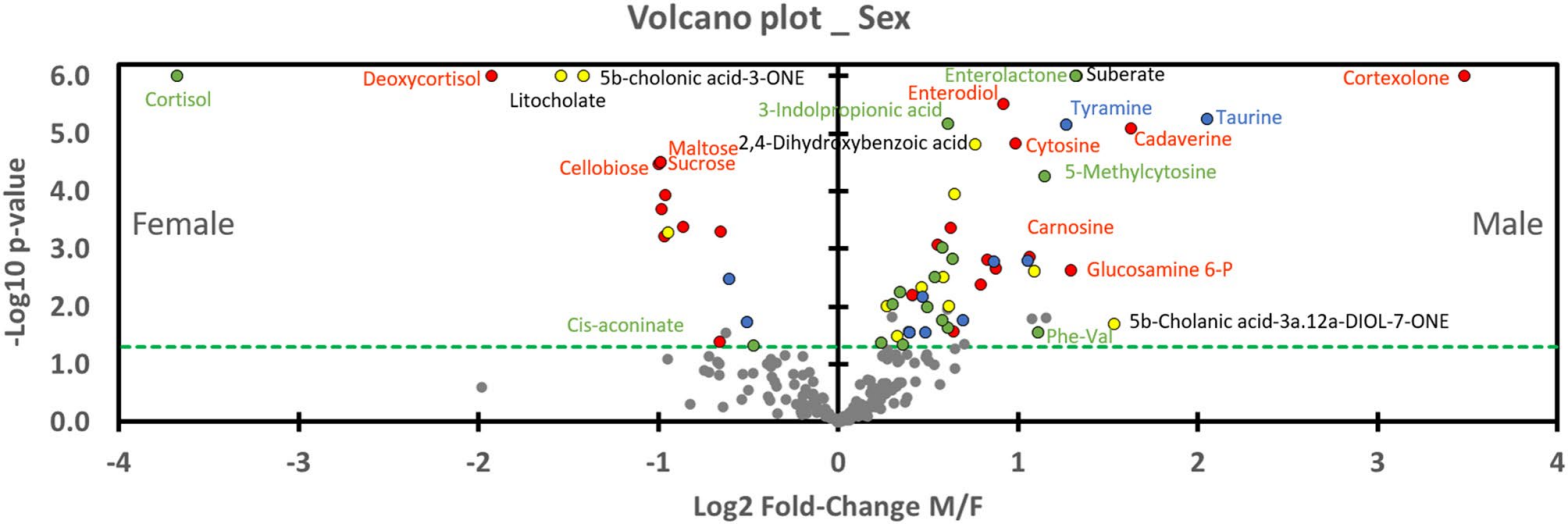
Volcano plot showing metabolomic data in rats (n = 57) according to the gender. Fecal metabolites over-expressed in males are on the right and those of female rats are on the left. The dashed line shows where *p* = 0.05 with points above the line having *p* < 0.05 and points below the line having *p* > 0.05. Colors of the dots are in accordance with the Venn diagram and whose significance is true according to the 2-way ANOVA. Red dots are for Sex*Strain*Interaction, for example. Grey dots are not significant according to the 2-way ANOVA and the post-hoc testes.

Graph 4 (Volcano Plot, Fig. 4) shows the level of metabolite expression in rat feces by sex. Metabolites located on the left (n = 17) of the vertical axis are significantly (*p* < 0.05) higher in females and those on the right are lower in males (n = 46). We emphasized that all metabolites except two influenced by Sex*Strain (16 green dots) are greater in males. Among these two exceptions, Cis-aconitate has a *p*-value near 0.05 and a low fold-change, while cortisol (in the upper left corner) has a very significant p-value and a great fold-change. Concretely, cortisol levels are higher in Std females but they decrease with resistance to DCS to approximate males (Std and Res). Red dot on its immediate right closeness represents another product of the cortisol metabolism: 21-deoxycortisol. 21-deoxycortisol levels are higher in Standard females but they decrease with resistance to approximate males. This last decreased between Std and Res animals but in a lesser extent. Then, both yellow dot (Sex) on the right of cortisol metabolites, are litocholate and 5b-cholanic acid -3-one.

In the upper right corner, cortexolone has higher fold-change ratio and p-value. On its left, dots of suberate (yellow; Sex) and enterolactone (green; Sex*Strain) are overlapped. Enterodiol (red) is just below, on the p-value scale. Blue points (Sex*Interaction) below mark aminoacid (Taurine and tyramine).

In order to access enrichment statistics and to analyze differential expression rates, we submitted these results to the ChemRICH database^27^ (www.chemrich.fiehnlab.ucdavis.edu). 226 compounds accompanied by their variation factor and their p-values were uploaded to the ChemRICH server. 219 compounds were accepted. We analyzed the effects of the sex and those linked to the strain resistance. The ChemRich graphics aggregate metabolites that are significantly altered (depending on gender or resistance) according to their chemical similarity.

**Figure 5:**
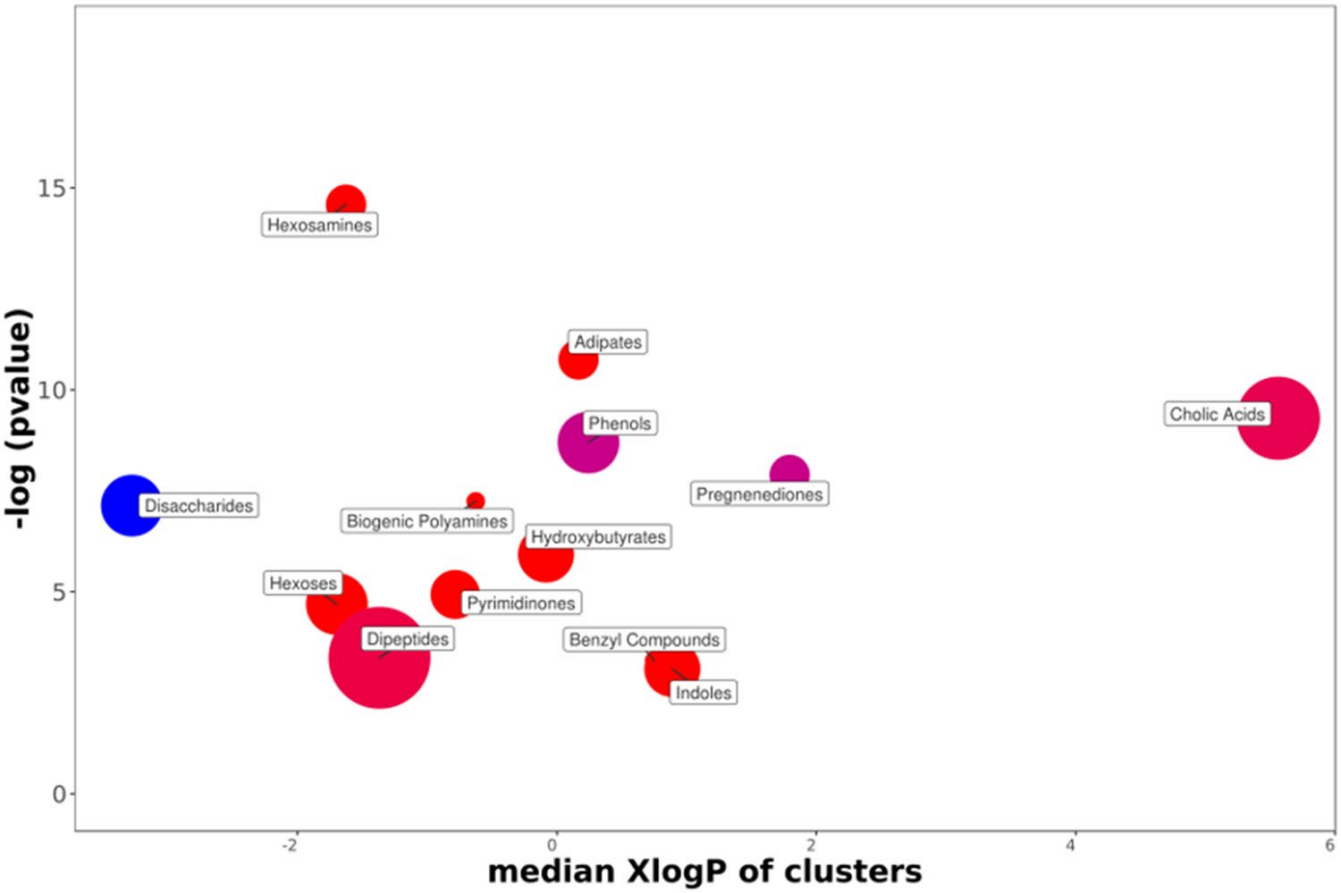
ChemRICH diagram according to the gender. Each disc reflects a significantly altered family of metabolites. These groups are developed from chemical similarities highlighted by a hierarchical Tanimoto map (not shown) accessible in the ChemRICH program. Enrichment *p*-values are given by the Kolmogorov–Smirnov test and displayed along the ordinate, with P values transformed in –log10. Disc sizes varies with the total number of metabolites. Red discs present groups of overexpressed metabolites in males while blue ones show overexpressed compounds in Females. Purple color represents both increased and decreased metabolites. For example, more adipate is found in the feces of male rats compared to females. Metabolite families are also scattered according to their increasing hydrophobicity (or decreasing hydrophilicity), from left to right along the abscissa (expressed as log P value).

The ChemRich graph (Fig. 5) based on gender shows a global overexpression (red circles) in the quantity of cholic acids (8 of the 12 identified compounds are altered) in males. The key compound is litocholate (a bile acid which is, however, underexpressed in males). Dipeptides are also overexpressed in the feces of male rats. Also noteworthy is the group of hexosamines (4/4 overexpressed in males), the key compound of which is glucosamine, useful in glycosylation. There is an underexpression of disaccharides (5/7) in males compared to females. Concerning pregnenediones, the 4 (out of 4) metabolites analyzed are significantly altered. This group includes cortisol and its derivate (21-deoxycortisol), both overrepresented in females but whose amounts decrease over generations, and also phytohormones overexpressed in males (17a-Hydroxyprogesterone and cortexolone). Cortexolone, an inhibitor of testosterone receptors, is particularly overexpressed in males with a fold-change of approximately 11. It decreases in amount in resistant individuals to approach the stable values of females. In another red circle in the graphic, it can be seen that enterodiol, a phytoestrogen from the benzyl compound family, is overexpressed in males. Enterodiol concentration profiles are similar to those of cortexolone.

Regarding phenols, 2 of the 7 metabolites analyzed are significantly altered. The first, presented by the ChemRich database as a key derivative, is 4-aminophenol. The other compound is 2- aminophenol. Both are overexpressed in males. Also in this family, phloretin was initially overexpressed in males, while tyrosol was initially overexpressed in females: they decreased with resistance to DCS.

### Metabolomic analysis on DCS-resistance

**Figure 6:**
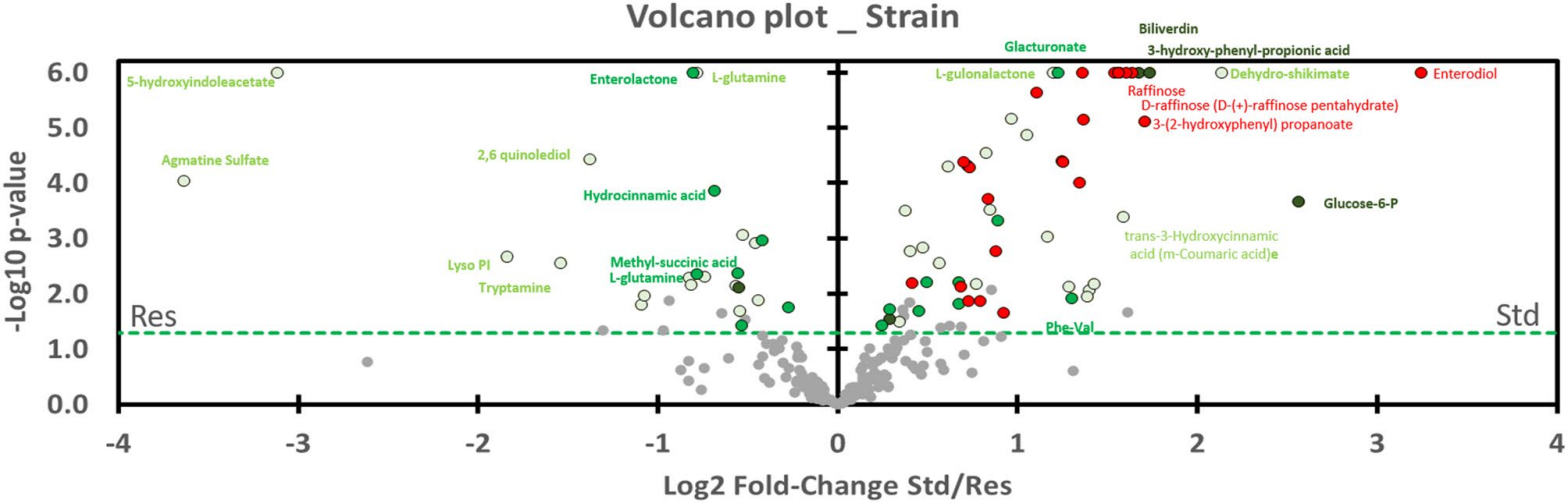
Volcano plot showing metabolomic data in rats (n = 57) according to the resistance of the strain. Fecal metabolites over-expressed in Std are on the right and those of the Res are on the left. The dashed line (green) shows where *p* = 0.05 with points above the line having *p* < 0.05 and points below the line having *p* > 0.05. Colors of the dots are in accordance with the Venn diagram and whose significance is true according to the 2-way ANOVA.

According to the Volcano Plot dedicated to the resistance to DCS (Fig. 6), 57 metabolites appearing on the right of the graph are significantly under-expressed in Res group compared to the Std animals while 24 other metabolites (on the left) are increased in resistant individuals. All the red dots (Sex*Strain*Interaction) are located to the right of the graph, which implies that they are expressed more in standard than in DCS-resistant animals. These red values of males and females converge on the downside with resistance to DCS. The metabolite which decreases the most in proportion, in the right upper corner (in red), and which diminished according to Sex*Strain*Interaction is enterodiol. On its left, glucose-6-P is in dark green, followed by dehydro-shikimate (light green), 3-hydroxyphenyl propionic acid (dark green), 3-(2-hydroxyphenyl)propanoate (red), biliverdin (dark green), and then 2 variant of raffinose (red). The red dots (Sex*Strain*Interaction) are more or less evenly distributed on both sides of the axis.

On the opposite, from the left to the right (in light green), agmatine sulfate, 5-hydroxyindoleacetate, lysoPI(16:0) (palmitoyl lysophosphatidylinositol), tryptamine and 2,6 quinolediol increase with resistance to DCS. Enterolactone is the upper dot in green on the left of the graph which is link to the Strain*Sex effect.

We then set up and trained a random forest classification (machine learning) on whole data (Sampling: random with replacement; method: random input; number of trees: 300; Sample size: 30; Min Node: 2; Max depth: 25; Mtry: 5; Cp: 0.0001) in order to extracted most contributing compounds for the description of breeding, i.e. resistance. We have chosen the algorithm and the hyperparameters with the lowest error rate. The Out-Of-Bag (OOB), i.e. error rate, value of random forest was of 3.51% and the algorithm predicted correctly, according to the confusion matrix, 95.1% of observed classes. We have chosen to select the first ten compounds, that is to say those having the greatest importance in the algorithm. The compounds selected in decreasing order of importance are: 5-hydroxyindolacetate, enterodiol, sucrose, 2,6-quinolinediol, 3-dehydroshikimate, galacturonate, azelate, maltose, agmatine sulfate, L-gulonolactone. The other algorithms presented slightly different results depending on the settings of the hyperparameters, but in the end 8 compounds come back redundantly. 5-hydroxyindolacetate and enterodiol had the greatest importance values in the algorithm.

**Figure 7:**
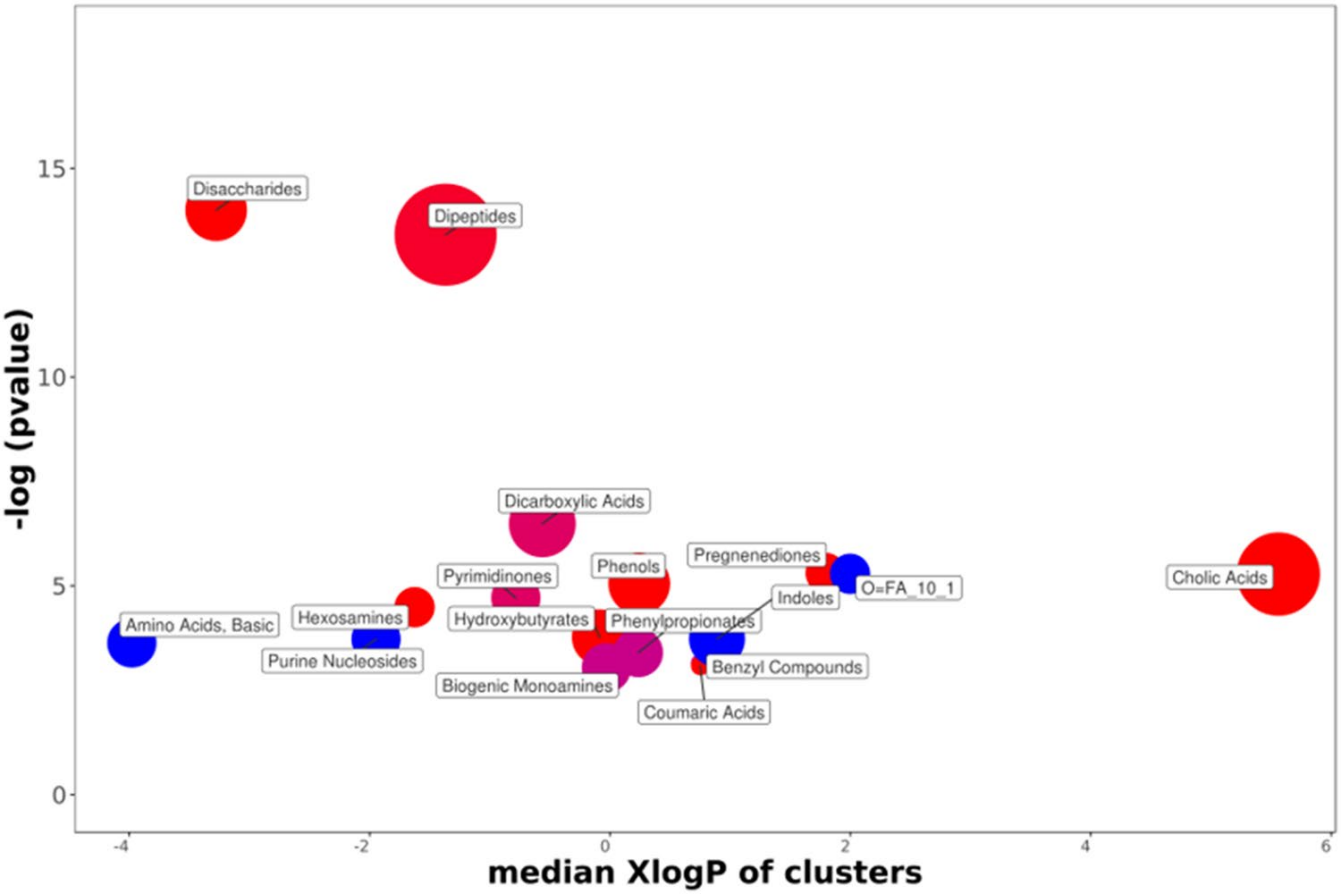
ChemRICH diagram according to the strain resistance. Each disc reflects a significantly altered family of metabolites. These groups are developed from chemical similarities highlighted by a hierarchical Tanimoto map (not shown) accessible in the ChemRICH program. Enrichment *p*-values are given by the Kolmogorov–Smirnov test. Disc sizes varies with the total number of metabolites. Blue or red discs present groups of overexpressed or underexpressed metabolites in Res compared to Std animals, respectively. Purple color represents both increased and decreased metabolites. For example, there are less disaccharides in the feces of the DCS-resistant rats.

The ChemRich graph (Fig. 7 + supp data 2 for details) based on resistance of strains shows a global underexpression in Resistant individuals compared to non-selected ones (red circles) in the quantity of cholic acids (3 compounds are significant). Dipeptides (9 decreased on 10 significantly altered, with N-acetyl asparagine as key compound) disaccharides (7/7 decreased, with sucrose as key compound) pregnenediones (cortisol, 21 deoxycortisol, cortexolone) hexosamines (with glucosamine and glucosamine 6-P) hydroxybutyrate and phenols (including phloretin and tyrosol) are underrepresented in Res groups.

In contrast, overexpressions (blue disc) are visible in the feces of DCS-resistant animals for 2 basic amino acids (L-glutamine and asparagine), 2 purines nucleosides (guanosine and inosine), and 2 dicarboxylic acids (denoted 0 = FA in the graph, including azelate and undecanedioic acid).

Enterodiol, the phytoestrogen of the benzyl compound family (purple circle), is overexpressed in Std rats, while enterolactone is overexpressed in Res animals. In the biogenic monoamine cluster (purple circle) tryptamine increases while histamine decreases in DCS-resistant animals.

## Discussion

### Influence of DCS-resistance

The DCS-resistant animal breeding program resulted in a strain of Wistar rats that was 3 times less likely to develop signs suggestive of DCS^9,10^, independently from any preconditioning strategy or diving acclimatization. This unique model of resistance to DCS makes therefore possible the study its physiological background, and particularly its fecal metabolome from caecum, associated with resistance to DCS by comparing these “spontaneous” DCS-resistant Wistar rats with standard non-selected ones. The main result of this analysis is a difference between the standard and DCS-resistant rats in their fecal composition from caecum.

Sex was previously described as a factor contributing to DCS^7,8^. Our AHC analysis also confirms differences between males and females in the non-selected animals but it fades in the rats selected for their resistance to DCS. This concerns 63 metabolites, 25 of which are unrelated to resistance. Although it may be of interest elsewhere, the differences in the metabolome linked only to sex, and therefore not linked to selection, does not represent the objective of this work. The significant influences of Sex, Sex*Interaction, and also pure Interaction, will not be discussed here. By postulating that the fruit of the breeding is the result of the selection of rats resistant to DCS, we can consider that 81 fecal compounds are related to this phenotypical outcome. Of course, some of them (n = 39) are also influenced by sex. This confirms that a sex-related fecal metabolism may also be linked to a phenotype at risk for DCS. This implies that the expression of these metabolites must be able to be influenced by the presence of the genome linked to X or Y, directly or indirectly. This should be in the 16 (Sex*Strain) and 23 (Sex*Strain*Interaction) compounds. While the analysis is obviously not exhaustive, these 39 compounds represent an important part of the 81 substances whose level of expression has been modified by breeding. There may also be mutual, or cascading, interactions among all these metabolites that alter their respective levels of expression. For the other compounds (36 Strain and 6 Strain*Interaction), it must also be possible to suggest that their content, although being able to be modulated elsewhere, must also be modulated by the autosomal part of the genome. The gut microbiota has not yet been discussed, but obviously it must have a role in the expression of fecal metabolites.

### Cecal content of metabolites linked to the breeding

Of the 81 metabolites linked to breeding, 39 are sex-linked and 23 of which are synergistically modulated.

These 23 compounds (in red, Sex*Strain*Interaction) present a homogeneous group effect with a general downward trend in DCS-resistant rats, even if this drop is sometimes true only in one sex. Specifically, it can be said that none of these 23 metabolites significantly increase in Res group and that the male and female profiles (the variation of which sometimes affects only one sex) converge in individuals resistant to DCS, which gives an average downward trend.

The male and female profiles of the 16 compounds (in green, Sex*Strain) also tend to converge in DCS-resistant groups except for 2 metabolites which diverge: enterolactone and methyl succinic acid.

Concerning the 36 Strain and the 6 Strain*Interaction compounds, one cannot apply a general tendency to increase or decrease, but only an aptitude to vary with the selection breeding.

### Expression of metabolites essentially linked to the microbial community

We found 8 metabolites specific to the gut microbial community that are also under pressure from selection.

Among them, enterodiol (Sex*Strain*Interaction) _highlighted as a key factor in the algorithm generated by the random forest classification_ enterolactone (Sex*Strain) and phloretin (Strain) are described to be endocrine-disrupting chemicals, or as phytoestrogen, because they are thought to interfere with estrogen metabolism in animals and humans^28^. Although not specific of the microbial community, another sexual hormone, cortexolone (Sex*Strain*Interaction) which is androgen antagonist, decreased. These compounds could participate in blurring the difference between the sexes. Their role is still unclear, while inhibitory actions on aromatase^29^ or blocking cell-membrane hormones-receptors were reported^30^. These three gut-microbiota products have also weak antioxidant activity^31^ like another compound azelate, a byproduct of pityrosporum fungal mycelia metabolism^32^.

Trehalose^33^ as shikimate and its derivative 3-dehydroshikimate^34^, the three being down-regulated in DCS-resistant individuals, are produced by plants yeast, fungi, insects, invertebrates and bacteria but never by animals. The polyamine cadaverine also comes from colonic bacteria^35^. They all decreased with resistance to DCS.

As the diet was the same for standard animals and selected animals during at least 2 weeks before the sampling of cecal content, it is likely that changes in the fecal composition come from a reorganization of the activity of the intestinal microbiota community.

### Metabolites indicative of a reshuffle of the DCS-resistant animals

A change in fecal composition may result from the rearrangement of the microbial community in relation to the change in the physiological characteristics of the host. The host has the ability to modulate the development of microbial populations through a number of chemical compounds with antibiotic or anti-fungal properties^36^. Self-regulation of the different microbial communities is also true^37^. Thus, we notice in animals resistant to DCS an increase in 2,6-quinolediol^38^ and Azelate^32^, while m-coumaric acid, i.e. trans-3-hydroxycinnamic acid (3-coumarate) et 4-coumarate^39,40^, decrease, all compounds with antibiotic properties.

This change could reveal a deeper host metabolic shuffle. The composition of the gut microbiota is strongly impacted by the genetic background of the animal strain^11^.

Concretely, we recorded a new shape in hormonal (steroid) profile including decrease in cortisol (glucocorticoid), 21-deoxycortisol^41^, and also cortexolone a precursor of cortisol when catalyzed by the enzyme cytochrome P450^42^. There is a concomitant change in the energy metabolism of sugars, energy metabolism in general, which results in decrease of xylose, sucrose, raffinose, cellobiose, trehalose^43^ or of glucose-6-phosphate^44^. The decrease in glucose-6-phosphate could be linked to the increase in 6-aminopyridine-3-carboxylic acid (Strain*Interaction), also known as 6-aminonicotinamide (6-AN). It is used in order to disrupt NADPH production in the pentose phosphate pathway^45–47^. This compound is metabolized into an analog of NADP + , thus competitively inhibiting the critical NADPH-producing enzymes 6-phosphogluconate dehydrogenase (PGD) and glucose 6-phosphate dehydrogenase (G6PD)^48^. Interestingly, a study linking the oxidative stress to the pentose phosphate pathway demonstrated that its inhibition through 6-AN has improved the oxidative stress response^49^. Oxidative stress has been regularly evoked in diving^21,50–53^. Elsewhere, the decreases in gulonolactone galacturonate and glucuronate may also be of interest for their link to the vitamin C pathway and its antioxidative properties^54^. The decrease in xanthine^55,56^ and tyrosol^57,58^ in DCS-resistant rats could also be interpreted as an antioxidant response reshuffle. Another overexpressed metabolite in these animals, undecanedioic acid, in an analysis conducted in urine has been linked to the mitochondrial activity^59^, or else it could be linked to a higher proportion of bacteria in the caecum^60^.

Furthermore, noted in DCS-resistant animals is decrease expression of cholic acids (ChemRich Fig. 7), chenodeoxycholate deoxycholate and hyodeoxycholic (Sex*Strain*Interaction), which are biliary acids or their co-products, the function of which is to help absorb dietary fats by the formation of micellae. An increase in 16-0-lysoPI (lysophopsholipids) is actually noted in feces from individuals resistant to DCS. Their second role is to inhibit (by an antiseptic or even antibiotic effect) the proliferation of the bacteria in the upper part of the digestive tract. However, a larger role is now attributed to biliary salts throughout the body^61^.

5-hydroxyindoleacetate or 5HIAA (Strain) was highlighted as a key factor in the algorithm generated by the random forest classification. It is overexpressed in DCS-resistant individuals and we wonder considering its place in the tryptophan pathway (from indole; cf ChemRich Analysis) and therefore in the metabolism of serotonin if it could have an influence on the colon mobility^62^ but serotonin level is not altered (supp data 1). This does not exclude that serotonin turnover, as indicated by the 5-HIAA/5HT ratio could be altered^63^. Hence, we conducted a Mann–Whitney analysis and noticed a very significant difference between standard and selected rats ratio (*p* < 0.000001). This ratio is tenfold higher in selected animals (Figure in Supp data 3). The serotonergic system has been recognized for decades as an important signaling molecule in the gut^62^, not just locally for its role on the gastrointestinal tract, but because it is clearly integrated in the brain-gut axis concept^63^. We lack evidence to evoke some mechanism in this study but this serotonergic dysregulation deserves attention, because the enzymes of these pathways are immune and stress responsive^63^. This remark should be put in parallel with the decrease in cortisol mentioned above. We could also have evoked a change due to the metabolism of tryptophan but its quantities do not change significantly (not shown).

The breeding of DCS-resistant rats did operate a reshuffle of the metabolome, including hormonal and energetic regulation, whether in the host or in the microbial community. Concerning metabolites exposed in this work, some compounds could be linked to sexual susceptibility-to-DCS, but the selection over generation participate in blurring the difference between the sexes.

### Moderation

This work obviously has not analyzed all the metabolites composing the faeces, in terms of pure technical analysis or in terms of discussion of each of the compounds identified. It is therefore possible these missing metabolites may play a determinant role.

We wrote that a certain number of cecal compounds are linked to DCS-resistant strains and that may also may be link to the sex. However, as this work study fecal metabolome from caecum, it is not possible to exclude the fact that another diet could have an influence.

We assume that the diets of animals may differ very slightly, and that a two-week adaptation period may seem a little bit short regarding the microbiote community. This could induce inflammation and thus make the animals more susceptible to DCS^23^. However, this does not seem sufficient to explain the increase in resistance over generations. At least, we had not demonstrated a difference in susceptibility to DCS in rats receiving two different diets^23^.

We have mentioned “under” or “overexpression” suggesting a higher or a less absorption of metabolites, but it needs to be reminded that the compounds identified here are the result of the exogenous digestion (food bolus) and the endogenous digestion (including among other things debris from cells of the digestive system) substrates, which are themselves concerned with microbial activity, and that as such it remains difficult to identify their origin correctly. Characterizing the microbial community would partially clarify this point.

Although our results highlight various differences between DCS-resistant and non-resistant animals we have not yet experimentally questioned the relationship between these metabolomic changes and resistance to DCS. It is therefore probable that not all of these differences are directly related to DCS resistance and that some may represent collateral modifications only. This is the subject of continued experimentation by our research group and others.

From the point of view of clinical practice, it would be interesting to assess how much the reshuffle of the fecal metabolome affects the risk of DCS, and consequently whether a resetting of the Res metabolome into the Std one could be provocative. As a result, it could also impact DCS therapeutic with the aim to avoid degrading microbiota community, by limiting the side effects of hyperbaric oxygen therapy on anerobic species for example. At the same time, it should be reminded inter-species similarities of the gut microbiota (e.g. between human and rats) to make known that such a study can be useful to humans^64,65^.

## Conclusion

This study shows for the first time the spectral fingerprint of the fecal metabolome from caecum of a strain of rats resistant to decompression sickness. On the one hand, statistical analysis shows a panel of 81 compounds whose expression had changed following the selection of rats based on their resistance to DCS. These results suggest hormonal and energetic reshuffle, including steroids sugars or antibiotic compounds, whether in the host or in the microbial community. On the other hand, this study also confirms a difference between males and females in the metabolome of non-selected rats, which disappear with selective breeding. As metabolomic studies cannot be exhaustive, additional investigations must check each point evoked and its clinical relevance.

## Supplementary Information

Supplementary Information.
