# Supplementary material for: Evidence of a hormonal reshuffle in the cecal metabolome fingerprint of a strain of rats resistant to decompression sickness
Source: Sci Rep. 2021 Apr 15;11:8317. doi: 10.1038/s41598-021-87952-y (PMC8050073; doi:10.1038/s41598-021-87952-y)

**Supplementary Data**

**Evidence of a hormonal reshuffle in the cecal metabolome fingerprint of a strain of rats resistant to decompression sickness**

Nicolas VALLEE, Emmanuel DUGRENOT, Anne-Virginie DESRUELLE, Catherine TARDIVEL, Jean-Charles MARTIN, Anthony GUERNEC, Alain BOUSSUGES, Sarah RIVES, Jean-Jacques RISSO, François GUERRERO.


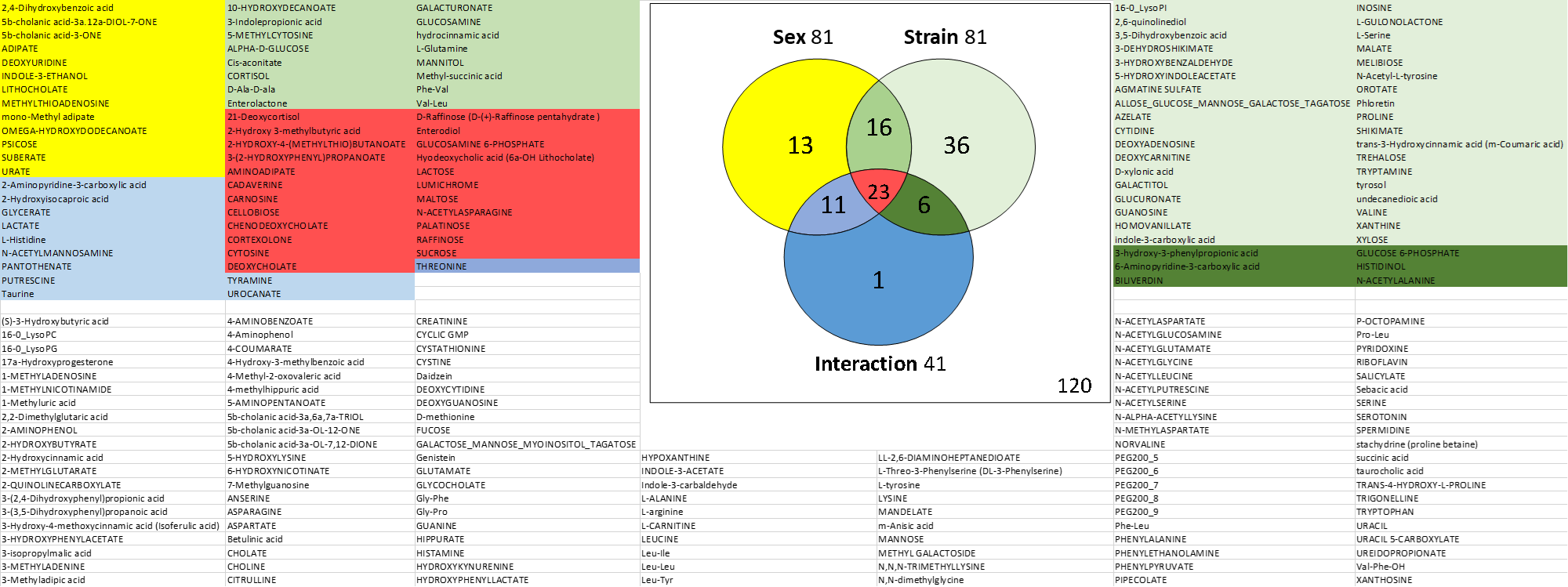


**Supp Data 1** Details of fecal metabolites altered according the sex and the resistance of the strain to DCS. 226 compounds have been analyzed.

|  | **Metabolite** | **Pr > F** | **p-value Sex** | **Fold Change (M/F)** | **p-value Strain** | **Fold Change Std/Res** | **p interaction** |
| --- | --- | --- | --- | --- | --- | --- | --- |
| Se | 5b-cholanic acid-3-ONE | 0,000 | 0,0000 | 0,37 | 0,1770 | 0,81 | 0,7939 |
| Se | LITHOCHOLATE | 0,000 | 0,0000 | 0,34 | 0,0810 | 0,76 | 0,0872 |
| Se | SUBERATE | 0,000 | 0,0000 | 2,51 | 0,2664 | 1,15 | 0,1220 |
| Se | 2,4-Dihydroxybenzoic acid | 0,000 | 0,0000 | 1,69 | 0,6421 | 1,05 | 0,8731 |
| Se | OMEGA-HYDROXYDODECANOATE | 0,001 | 0,0001 | 1,56 | 0,3614 | 1,10 | 0,0905 |
| Se | ADIPATE | 0,003 | 0,0031 | 1,50 | 0,5346 | 1,08 | 0,2768 |
| Se | URATE | 0,005 | 0,0005 | 0,52 | 0,8553 | 0,97 | 0,5842 |
| Se | mono-Methyl adipate | 0,016 | 0,0326 | 1,26 | 0,1418 | 0,86 | 0,2810 |
| Se | PSICOSE | 0,016 | 0,0098 | 1,21 | 0,1393 | 1,11 | 0,7438 |
| Se | INDOLE-3-ETHANOL | 0,018 | 0,0024 | 2,13 | 0,4885 | 1,17 | 0,0615 |
| Se | DEOXYURIDINE | 0,022 | 0,0047 | 1,38 | 0,1612 | 1,17 | 0,4686 |
| Se | 5b-cholanic acid-3a.12a-DIOL-7-ONE | 0,036 | 0,0201 | 2,89 | 0,2183 | 0,60 | 0,7772 |
| Se | METHYLTHIOADENOSINE | 0,045 | 0,0099 | 1,53 | 0,7814 | 1,04 | 0,9206 |
| Str | 3-DEHYDROSHIKIMATE | 0,000 | 0,7489 | 0,96 | 0,0000 | 4,39 | 0,6568 |
| Str | 5-HYDROXYINDOLEACETATE | 0,000 | 0,2373 | 0,79 | 0,0000 | 0,12 | 0,1428 |
| Str | AZELATE | 0,000 | 0,8715 | 1,01 | 0,0000 | 0,58 | 0,6833 |
| Str | L-GULONOLACTONE | 0,000 | 0,0695 | 1,24 | 0,0000 | 2,32 | 0,2236 |
| Str | MALATE | 0,000 | 0,6755 | 1,05 | 0,0000 | 2,30 | 0,4075 |
| Str | DEOXYCARNITINE | 0,000 | 0,0809 | 0,77 | 0,0000 | 2,07 | 0,2337 |
| Str | TREHALOSE | 0,000 | 0,6388 | 1,06 | 0,0000 | 1,95 | 0,3573 |
| Str | MELIBIOSE | 0,000 | 0,5785 | 1,07 | 0,0000 | 1,77 | 0,9535 |
| Str | trans-3-Hydroxycinnamic acid (m-Coumaric acid) | 0,000 | 0,0715 | 0,61 | 0,0004 | 3,00 | 0,0661 |
| Str | 2,6-quinolinediol | 0,000 | 0,9168 | 1,02 | 0,0000 | 0,38 | 0,3679 |
| Str | OROTATE | 0,001 | 0,2130 | 1,21 | 0,0003 | 1,80 | 0,2473 |
| Str | GLUCURONATE | 0,001 | 1,0000 | 1,00 | 0,0001 | 1,53 | 0,4515 |
| Str | AGMATINE SULFATE | 0,001 | 0,9423 | 1,03 | 0,0001 | 0,08 | 0,8295 |
| Str | undecanedioic acid | 0,002 | 0,0714 | 1,19 | 0,0012 | 0,73 | 0,7308 |
| Str | SHIKIMATE | 0,002 | 0,3924 | 0,94 | 0,0003 | 1,30 | 0,4053 |
| Str | DEOXYADENOSINE | 0,003 | 0,1435 | 0,72 | 0,0009 | 2,25 | 0,5337 |
| Str | D-xylonic acid | 0,005 | 0,0567 | 1,21 | 0,0015 | 1,39 | 0,4001 |
| Str | tyrosol | 0,006 | 0,1369 | 0,61 | 0,0065 | 2,68 | 0,0540 |
| Str | 16-0_LysoPI | 0,006 | 0,4630 | 1,30 | 0,0021 | 0,28 | 0,1785 |
| Str | L-Serine | 0,010 | 0,7321 | 0,97 | 0,0009 | 0,69 | 0,9721 |
| Str | XYLOSE | 0,010 | 0,1874 | 1,12 | 0,0017 | 1,32 | 0,2110 |
| Str | PROLINE | 0,010 | 0,1501 | 0,84 | 0,0132 | 0,74 | 0,2285 |
| Str | indole-3-carboxylic acid | 0,011 | 0,5017 | 0,88 | 0,0068 | 0,57 | 0,1509 |
| Str | TRYPTAMINE | 0,014 | 0,1546 | 0,63 | 0,0027 | 0,34 | 0,7483 |
| Str | XANTHINE | 0,018 | 0,2617 | 1,15 | 0,0028 | 1,48 | 0,2636 |
| Str | 3,5-Dihydroxybenzoic acid | 0,021 | 0,1292 | 0,60 | 0,0084 | 2,64 | 0,5466 |
| Str | 3-HYDROXYBENZALDEHYDE | 0,022 | 0,2463 | 1,25 | 0,0051 | 0,56 | 0,6111 |
| Str | INOSINE | 0,024 | 0,0993 | 0,63 | 0,0106 | 0,47 | 0,8853 |
| Str | CYTIDINE | 0,025 | 0,0948 | 0,79 | 0,0072 | 0,67 | 0,5532 |
| Str | N-Acetyl-L-tyrosine | 0,031 | 0,4486 | 1,14 | 0,0048 | 0,60 | 0,9105 |
| Str | VALINE | 0,033 | 0,9319 | 1,01 | 0,0205 | 0,69 | 0,1025 |
| Str | GUANOSINE | 0,035 | 0,3601 | 0,76 | 0,0157 | 0,47 | 0,3396 |
| Str | HOMOVANILLATE | 0,040 | 0,7222 | 0,89 | 0,0112 | 2,62 | 0,2387 |
| Str | GALACTITOL | 0,040 | 0,3326 | 1,20 | 0,0067 | 1,70 | 0,9970 |
| Str | Phloretin | 0,046 | 0,4773 | 1,24 | 0,0074 | 2,44 | 0,2835 |
| Str | ALLOSE_GLUCOSE_MANNOSE_GALACTOSE_TAGATOSE | 0,049 | 0,0815 | 1,21 | 0,0316 | 1,27 | 0,9506 |
| Se*Str | CORTISOL | 0,000 | 0,0000 | 0,08 | 0,0151 | 1,60 | 0,1035 |
| Se*Str | Enterolactone | 0,000 | 0,0000 | 2,50 | 0,0000 | 0,57 | 0,1279 |
| Se*Str | GALACTURONATE | 0,000 | 0,0458 | 1,28 | 0,0000 | 2,34 | 0,3600 |
| Se*Str | 3-Indolepropionic acid | 0,000 | 0,0000 | 1,53 | 0,0011 | 0,75 | 0,1974 |
| Se*Str | hydrocinnamic acid | 0,000 | 0,0010 | 1,50 | 0,0001 | 0,62 | 0,7936 |
| Se*Str | 5-METHYLCYTOSINE | 0,000 | 0,0001 | 2,21 | 0,0375 | 0,69 | 0,3233 |
| Se*Str | Methyl-succinic acid | 0,000 | 0,0101 | 1,41 | 0,0042 | 0,68 | 0,0818 |
| Se*Str | Val-Leu | 0,001 | 0,0174 | 1,49 | 0,0005 | 1,85 | 0,1052 |
| Se*Str | GLUCOSAMINE | 0,002 | 0,0015 | 1,55 | 0,0201 | 1,37 | 0,6615 |
| Se*Str | D-Ala-D-ala | 0,002 | 0,0031 | 1,45 | 0,0061 | 1,41 | 0,3182 |
| Se*Str | L-Glutamine | 0,002 | 0,0233 | 1,52 | 0,0044 | 0,58 | 0,1005 |
| Se*Str | Cis-aconitate | 0,004 | 0,0479 | 0,72 | 0,0060 | 1,59 | 0,0563 |
| Se*Str | 10-HYDROXYDECANOATE | 0,004 | 0,0091 | 1,23 | 0,0173 | 0,83 | 0,9638 |
| Se*Str | ALPHA-D-GLUCOSE | 0,004 | 0,0057 | 1,27 | 0,0191 | 1,22 | 0,9127 |
| Se*Str | Phe-Val | 0,017 | 0,0278 | 2,16 | 0,0119 | 2,47 | 0,3062 |
| Se*Str | MANNITOL | 0,030 | 0,0415 | 1,18 | 0,0373 | 1,18 | 0,0542 |
| I | THREONINE | 0,037 | 0,0833 | 1,27 | 0,0971 | 0,80 | 0,0429 |
| Se*I | Taurine | 0,000 | 0,0000 | 4,15 | 0,2259 | 1,35 | 0,0232 |
| Se*I | TYRAMINE | 0,000 | 0,0000 | 2,41 | 0,2870 | 1,20 | 0,0355 |
| Se*I | PUTRESCINE | 0,001 | 0,0283 | 1,40 | 0,0571 | 0,75 | 0,0014 |
| Se*I | PANTOTHENATE | 0,003 | 0,0033 | 0,66 | 0,1102 | 1,25 | 0,0087 |
| Se*I | 2-Hydroxyisocaproic acid | 0,006 | 0,0016 | 2,08 | 0,1107 | 1,41 | 0,0405 |
| Se*I | L-Histidine | 0,011 | 0,0017 | 1,82 | 0,7535 | 0,95 | 0,0411 |
| Se*I | N-ACETYLMANNOSAMINE | 0,013 | 0,0067 | 1,38 | 0,0959 | 1,21 | 0,0263 |
| Se*I | LACTATE | 0,035 | 0,0174 | 1,62 | 0,3653 | 1,19 | 0,0165 |
| Se*I | GLYCERATE | 0,036 | 0,0182 | 0,70 | 0,2924 | 1,16 | 0,0461 |
| Se*I | 2-Aminopyridine-3-carboxylic acid | 0,042 | 0,0275 | 1,32 | 0,4747 | 0,92 | 0,0219 |
| Se*I | UROCANATE | 0,042 | 0,0275 | 1,32 | 0,4747 | 0,92 | 0,0219 |
| Str*I | BILIVERDIN | 0,000 | 0,1082 | 0,77 | 0,0000 | 3,19 | 0,0330 |
| Str*I | 3-hydroxy-3-phenylpropionic acid | 0,000 | 0,1993 | 0,78 | 0,0000 | 3,33 | 0,0268 |
| Str*I | HISTIDINOL | 0,000 | 0,0712 | 0,82 | 0,0000 | 1,65 | 0,0076 |
| Str*I | GLUCOSE 6-PHOSPHATE | 0,000 | 0,0819 | 0,52 | 0,0002 | 5,92 | 0,0225 |
| Str*I | 6-Aminopyridine-3-carboxylic acid | 0,004 | 0,2169 | 1,19 | 0,0077 | 0,68 | 0,0186 |
| Str*I | N-ACETYLALANINE | 0,013 | 0,4383 | 1,07 | 0,0285 | 1,22 | 0,0081 |
| Se*Str*I | 21-Deoxycortisol | 0,000 | 0,0000 | 0,26 | 0,0000 | 2,37 | 0,0011 |
| Se*Str*I | CADAVERINE | 0,000 | 0,0000 | 3,09 | 0,0001 | 2,54 | 0,0009 |
| Se*Str*I | CELLOBIOSE | 0,000 | 0,0000 | 0,50 | 0,0000 | 2,96 | 0,0000 |
| Se*Str*I | CORTEXOLONE | 0,000 | 0,0000 | 11,18 | 0,0002 | 1,79 | 0,0009 |
| Se*Str*I | CYTOSINE | 0,000 | 0,0000 | 1,98 | 0,0000 | 2,15 | 0,0028 |
| Se*Str*I | D-Raffinose (D-(+)-Raffinose pentahydrate ) | 0,000 | 0,0002 | 0,51 | 0,0000 | 3,11 | 0,0031 |
| Se*Str*I | Enterodiol | 0,000 | 0,0000 | 1,89 | 0,0000 | 9,47 | 0,0002 |
| Se*Str*I | LACTOSE | 0,000 | 0,0004 | 0,55 | 0,0000 | 2,91 | 0,0002 |
| Se*Str*I | MALTOSE | 0,000 | 0,0000 | 0,50 | 0,0000 | 2,94 | 0,0000 |
| Se*Str*I | N-ACETYLASPARAGINE | 0,000 | 0,0006 | 0,51 | 0,0000 | 2,58 | 0,0030 |
| Se*Str*I | PALATINOSE | 0,000 | 0,0005 | 0,63 | 0,0000 | 2,57 | 0,0013 |
| Se*Str*I | RAFFINOSE | 0,000 | 0,0001 | 0,51 | 0,0000 | 3,04 | 0,0010 |
| Se*Str*I | SUCROSE | 0,000 | 0,0000 | 0,50 | 0,0000 | 2,94 | 0,0000 |
| Se*Str*I | 3-(2-HYDROXYPHENYL)PROPANOATE | 0,000 | 0,0404 | 0,63 | 0,0000 | 3,26 | 0,0127 |
| Se*Str*I | AMINOADIPATE | 0,000 | 0,0004 | 1,54 | 0,0001 | 1,66 | 0,0048 |
| Se*Str*I | 2-HYDROXY-4-(METHYLTHIO)BUTANOATE | 0,000 | 0,0022 | 1,83 | 0,0000 | 2,38 | 0,0012 |
| Se*Str*I | LUMICHROME | 0,000 | 0,0008 | 1,47 | 0,0000 | 1,63 | 0,0037 |
| Se*Str*I | 2-Hydroxy 3-methylbutyric acid | 0,000 | 0,0042 | 1,73 | 0,0017 | 1,84 | 0,0021 |
| Se*Str*I | Hyodeoxycholic acid (6a-OH Lithocholate) | 0,000 | 0,0015 | 1,77 | 0,0073 | 1,61 | 0,0026 |
| Se*Str*I | CHENODEOXYCHOLATE | 0,001 | 0,0063 | 1,33 | 0,0063 | 1,33 | 0,0029 |
| Se*Str*I | CARNOSINE | 0,001 | 0,0014 | 2,09 | 0,0133 | 1,73 | 0,0096 |
| Se*Str*I | GLUCOSAMINE 6-PHOSPHATE | 0,003 | 0,0024 | 2,45 | 0,0223 | 1,90 | 0,0302 |
| Se*Str*I | DEOXYCHOLATE | 0,006 | 0,0272 | 1,56 | 0,0133 | 1,65 | 0,0113 |
| NS | (S)-3-Hydroxybutyric acid | 0,086 | 0,9909 | 1,00 | 0,4749 | 1,21 | 0,0207 |
| NS | 16-0_LysoPC | 0,059 | 0,2277 | 1,48 | 0,0462 | 0,51 | 0,3491 |
| NS | 16-0_LysoPG | 0,899 | 0,7119 | 1,06 | 0,5517 | 0,91 | 0,9075 |
| NS | 17a-Hydroxyprogesterone | 0,051 | 0,0067 | 1,33 | 0,5741 | 1,06 | 0,4945 |
| NS | 1-METHYLADENOSINE | 0,636 | 0,8541 | 0,97 | 0,2236 | 0,83 | 0,7946 |
| NS | 1-METHYLNICOTINAMIDE | 0,556 | 0,5010 | 0,56 | 0,5341 | 0,59 | 0,4453 |
| NS | 1-Methyluric acid | 0,481 | 0,2150 | 1,20 | 0,3856 | 0,88 | 0,7131 |
| NS | 2,2-Dimethylglutaric acid | 0,269 | 0,1946 | 1,13 | 0,3072 | 1,10 | 0,5782 |
| NS | 2-AMINOPHENOL | 0,194 | 0,0683 | 1,41 | 0,3937 | 0,85 | 0,2164 |
| NS | 2-HYDROXYBUTYRATE | 0,086 | 0,9816 | 0,99 | 0,4754 | 1,21 | 0,0209 |
| NS | 2-Hydroxycinnamic acid | 0,535 | 0,4261 | 0,77 | 0,6084 | 0,85 | 0,4561 |
| NS | 2-METHYLGLUTARATE | 0,162 | 0,2502 | 0,25 | 0,1666 | 0,16 | 0,3892 |
| NS | 2-QUINOLINECARBOXYLATE | 0,556 | 0,1633 | 0,77 | 0,9235 | 0,98 | 0,8165 |
| NS | 3-(2,4-Dihydroxyphenyl)propionic acid | 0,068 | 0,2789 | 0,71 | 0,0583 | 1,88 | 0,0968 |
| NS | 3-(3,5-Dihydroxyphenyl)propanoic acid | 0,349 | 0,4141 | 0,69 | 0,2661 | 1,67 | 0,2032 |
| NS | 3-Hydroxy-4-methoxycinnamic acid (Isoferulic acid) | 0,343 | 0,2517 | 1,14 | 0,1381 | 1,18 | 0,6961 |
| NS | 3-HYDROXYPHENYLACETATE | 0,835 | 0,5004 | 1,09 | 0,8861 | 1,02 | 0,7516 |
| NS | 3-isopropylmalic acid | 0,676 | 0,5576 | 1,06 | 0,4039 | 0,92 | 0,6749 |
| NS | 3-METHYLADENINE | 0,059 | 0,6872 | 1,04 | 0,0141 | 1,32 | 0,4273 |
| NS | 3-Methyladipic acid | 0,319 | 0,0681 | 1,25 | 0,8122 | 1,03 | 0,7034 |
| NS | 4-AMINOBENZOATE | 0,762 | 0,7125 | 0,97 | 0,3927 | 0,92 | 0,7363 |
| NS | 4-Aminophenol | 0,065 | 0,0111 | 1,52 | 0,3569 | 1,16 | 0,5277 |
| NS | 4-COUMARATE | 0,144 | 0,7955 | 1,12 | 0,0218 | 3,05 | 0,6521 |
| NS | 4-Hydroxy-3-methylbenzoic acid | 0,923 | 0,5525 | 1,09 | 0,8539 | 1,03 | 0,9532 |
| NS | 4-Methyl-2-oxovaleric acid | 0,059 | 0,9922 | 1,00 | 0,0292 | 0,70 | 0,1573 |
| NS | 4-methylhippuric acid | 0,374 | 0,6865 | 0,95 | 0,0943 | 1,25 | 0,9570 |
| NS | 5-AMINOPENTANOATE | 0,361 | 0,8762 | 0,97 | 0,9370 | 0,99 | 0,1143 |
| NS | 5b-cholanic acid-3a,6a,7a-TRIOL | 0,324 | 0,8768 | 0,97 | 0,5510 | 1,11 | 0,1013 |
| NS | 5b-cholanic acid-3a-OL-12-ONE | 0,034 | 0,2253 | 0,85 | 0,0693 | 1,29 | 0,1334 |
| NS | 5b-cholanic acid-3a-OL-7,12-DIONE | 0,248 | 0,0443 | 1,62 | 0,8301 | 1,05 | 0,4801 |
| NS | 5-HYDROXYLYSINE | 0,066 | 0,0938 | 1,34 | 0,8694 | 1,03 | 0,0105 |
| NS | 6-HYDROXYNICOTINATE | 0,377 | 0,4946 | 0,85 | 0,6379 | 0,90 | 0,2422 |
| NS | 7-Methylguanosine | 0,441 | 0,2044 | 1,26 | 0,3643 | 1,18 | 0,2703 |
| NS | ANSERINE | 0,155 | 0,0739 | 0,87 | 0,2041 | 1,10 | 0,3524 |
| NS | ASPARAGINE | 0,206 | 0,7399 | 0,87 | 0,0459 | 0,40 | 0,6772 |
| NS | ASPARTATE | 0,947 | 0,9723 | 1,01 | 0,5691 | 1,12 | 0,8288 |
| NS | Betulinic acid | 0,237 | 0,9932 | 1,00 | 0,1201 | 0,86 | 0,1790 |
| NS | CHOLATE | 0,065 | 0,0165 | 2,11 | 0,3341 | 0,75 | 0,6853 |
| NS | CHOLINE | 0,487 | 0,3240 | 1,19 | 0,3780 | 0,85 | 0,7093 |
| NS | CITRULLINE | 0,388 | 0,4297 | 0,92 | 0,4880 | 1,08 | 0,3144 |
| NS | CREATININE | 0,475 | 0,9275 | 1,03 | 0,2865 | 1,38 | 0,2418 |
| NS | CYCLIC GMP | 0,520 | 0,6781 | 0,87 | 0,2341 | 1,50 | 0,4307 |
| NS | CYSTATHIONINE | 0,335 | 0,4550 | 0,87 | 0,1633 | 1,30 | 0,3250 |
| NS | CYSTINE | 0,524 | 0,9226 | 1,04 | 0,1612 | 0,56 | 0,7076 |
| NS | Daidzein | 0,068 | 0,0883 | 1,42 | 0,0376 | 1,54 | 0,8907 |
| NS | DEOXYCYTIDINE | 0,251 | 0,9899 | 1,00 | 0,4038 | 0,77 | 0,0971 |
| NS | DEOXYGUANOSINE | 0,356 | 0,6631 | 0,87 | 0,7606 | 0,90 | 0,1571 |
| NS | D-methionine | 0,535 | 0,7645 | 1,03 | 0,8452 | 0,98 | 0,1580 |
| NS | FUCOSE | 0,854 | 0,5615 | 1,15 | 0,9370 | 1,02 | 0,4083 |
| NS | GALACTOSE_MANNOSE_MYOINOSITOL_TAGATOSE | 0,129 | 0,3135 | 1,13 | 0,1176 | 1,22 | 0,3164 |
| NS | Genistein | 0,107 | 0,0159 | 2,23 | 0,7982 | 0,92 | 0,4369 |
| NS | GLUTAMATE | 0,218 | 0,4852 | 0,90 | 0,0558 | 1,32 | 0,9445 |
| NS | GLYCOCHOLATE | 0,960 | 0,7756 | 1,11 | 0,7201 | 1,14 | 0,6759 |
| NS | Gly-Phe | 0,894 | 0,8248 | 0,96 | 0,9934 | 1,00 | 0,5447 |
| NS | Gly-Pro | 0,686 | 0,3824 | 0,94 | 0,4959 | 1,05 | 0,5195 |
| NS | GUANINE | 0,940 | 0,7921 | 1,08 | 0,7719 | 0,92 | 0,7159 |
| NS | HIPPURATE | 0,080 | 0,4120 | 0,82 | 0,0130 | 0,52 | 0,8743 |
| NS | HISTAMINE | 0,210 | 0,7888 | 0,95 | 0,0406 | 1,48 | 0,7519 |
| NS | HYDROXYKYNURENINE | 0,099 | 0,0152 | 1,23 | 0,4864 | 1,06 | 0,2996 |
| NS | HYDROXYPHENYLLACTATE | 0,320 | 0,0914 | 0,63 | 0,9655 | 1,01 | 0,9278 |
| NS | HYPOXANTHINE | 0,875 | 0,9094 | 1,01 | 0,4217 | 0,93 | 0,9481 |
| NS | INDOLE-3-ACETATE | 0,133 | 0,1191 | 1,57 | 0,1446 | 0,66 | 0,7453 |
| NS | Indole-3-carbaldehyde | 0,609 | 0,5249 | 1,14 | 0,3195 | 0,82 | 0,4838 |
| NS | L-ALANINE | 0,563 | 0,3175 | 1,18 | 0,6749 | 1,07 | 0,2036 |
| NS | L-arginine | 0,614 | 0,9299 | 1,04 | 0,2340 | 0,54 | 0,6176 |
| NS | L-CARNITINE | 0,853 | 0,8745 | 1,05 | 0,8450 | 0,94 | 0,4647 |
| NS | LEUCINE | 0,566 | 0,5242 | 1,08 | 0,6046 | 0,94 | 0,2098 |
| NS | Leu-Ile | 0,249 | 0,2425 | 1,24 | 0,3110 | 1,21 | 0,4302 |
| NS | Leu-Leu | 0,387 | 0,5935 | 1,18 | 0,1234 | 1,63 | 0,3647 |
| NS | Leu-Tyr | 0,058 | 0,5740 | 1,13 | 0,0084 | 1,81 | 0,8879 |
| NS | LL-2,6-DIAMINOHEPTANEDIOATE | 0,220 | 0,6182 | 0,93 | 0,0787 | 0,78 | 0,1974 |
| NS | L-Threo-3-Phenylserine (DL-3-Phenylserine) | 0,169 | 0,3456 | 0,87 | 0,0556 | 1,32 | 0,5081 |
| NS | L-tyrosine | 0,946 | 0,8894 | 1,02 | 0,7052 | 0,95 | 0,6622 |
| NS | LYSINE | 0,164 | 0,2668 | 1,15 | 0,2409 | 0,86 | 0,0789 |
| NS | MANDELATE | 0,300 | 0,6960 | 1,05 | 0,0676 | 0,81 | 0,9691 |
| NS | m-Anisic acid | 0,509 | 0,5478 | 0,64 | 0,2434 | 2,48 | 0,4291 |
| NS | MANNOSE | 0,136 | 0,3062 | 1,15 | 0,4250 | 1,12 | 0,1497 |
| NS | METHYL GALACTOSIDE | 0,229 | 0,0540 | 1,57 | 0,7366 | 0,93 | 0,2140 |
| NS | N,N,N-TRIMETHYLLYSINE | 0,224 | 0,0755 | 1,40 | 0,4774 | 0,88 | 0,9682 |
| NS | N,N-dimethylglycine | 0,555 | 0,5313 | 0,90 | 0,5314 | 0,90 | 0,4214 |
| NS | N-ACETYLASPARTATE | 0,257 | 0,5785 | 1,07 | 0,0683 | 1,25 | 0,7334 |
| NS | N-ACETYLGLUCOSAMINE | 0,081 | 0,0261 | 1,31 | 0,3188 | 1,12 | 0,0632 |
| NS | N-ACETYLGLUTAMATE | 0,107 | 0,6210 | 0,95 | 0,0193 | 1,28 | 0,7594 |
| NS | N-ACETYLGLYCINE | 0,797 | 0,4848 | 0,92 | 0,8162 | 0,97 | 0,7111 |
| NS | N-ACETYLLEUCINE | 0,254 | 0,1489 | 0,69 | 0,1967 | 1,39 | 0,4953 |
| NS | N-ACETYLPUTRESCINE | 0,050 | 0,2028 | 1,35 | 0,1922 | 0,74 | 0,0200 |
| NS | N-ACETYLSERINE | 0,268 | 0,2752 | 0,88 | 0,1435 | 1,18 | 0,7988 |
| NS | N-ALPHA-ACETYLLYSINE | 0,074 | 0,9535 | 0,99 | 0,0245 | 1,29 | 0,1665 |
| NS | N-METHYLASPARTATE | 0,173 | 0,1981 | 0,91 | 0,0972 | 1,13 | 0,3780 |
| NS | NORVALINE | 0,050 | 0,2094 | 1,27 | 0,5891 | 0,90 | 0,0645 |
| NS | PEG200_5 | 0,907 | 0,9996 | 1,00 | 0,4716 | 0,94 | 0,9183 |
| NS | PEG200_6 | 0,345 | 0,2203 | 1,09 | 0,6648 | 1,03 | 0,0947 |
| NS | PEG200_7 | 0,193 | 0,1356 | 0,90 | 0,9852 | 1,00 | 0,3575 |
| NS | PEG200_8 | 0,108 | 0,5846 | 0,95 | 0,0878 | 0,85 | 0,1868 |
| NS | PEG200_9 | 0,524 | 0,8601 | 0,99 | 0,5337 | 0,95 | 0,2514 |
| NS | Phe-Leu | 0,082 | 0,1003 | 1,45 | 0,0386 | 1,61 | 0,7692 |
| NS | PHENYLALANINE | 0,389 | 0,9127 | 0,99 | 0,4400 | 0,91 | 0,1775 |
| NS | PHENYLETHANOLAMINE | 0,235 | 0,8511 | 1,02 | 0,2761 | 0,87 | 0,1075 |
| NS | PHENYLPYRUVATE | 0,862 | 0,6984 | 0,92 | 0,6477 | 1,10 | 0,6652 |
| NS | PIPECOLATE | 0,054 | 0,0970 | 0,76 | 0,6057 | 0,92 | 0,1557 |
| NS | P-OCTOPAMINE | 0,969 | 0,6639 | 1,06 | 0,9407 | 0,99 | 0,9634 |
| NS | Pro-Leu | 0,074 | 0,2778 | 1,23 | 0,0222 | 0,64 | 0,8874 |
| NS | PYRIDOXINE | 0,749 | 0,3201 | 1,20 | 0,7428 | 0,94 | 0,5744 |
| NS | RIBOFLAVIN | 0,705 | 0,4095 | 1,18 | 0,5485 | 1,13 | 0,3513 |
| NS | SALICYLATE | 0,428 | 0,9478 | 0,99 | 0,1993 | 1,14 | 0,3607 |
| NS | Sebacic acid | 0,015 | 0,0720 | 1,18 | 0,1390 | 0,87 | 0,1208 |
| NS | SERINE | 0,845 | 0,5272 | 1,11 | 0,6789 | 0,93 | 0,5360 |
| NS | SEROTONIN | 0,448 | 0,7918 | 1,02 | 0,2349 | 1,10 | 0,2393 |
| NS | SPERMIDINE | 0,253 | 0,0896 | 1,26 | 0,3409 | 0,88 | 0,6844 |
| NS | stachydrine (proline betaine) | 0,165 | 0,0675 | 1,31 | 0,1468 | 1,23 | 0,4632 |
| NS | succinic acid | 0,275 | 0,9579 | 1,02 | 0,1810 | 1,48 | 0,1515 |
| NS | taurocholic acid | 0,210 | 0,3822 | 1,30 | 0,0717 | 1,75 | 0,7256 |
| NS | TRANS-4-HYDROXY-L-PROLINE | 0,547 | 0,3267 | 0,91 | 0,3257 | 0,91 | 0,9282 |
| NS | TRIGONELLINE | 0,683 | 0,7083 | 0,79 | 0,3699 | 0,56 | 0,6083 |
| NS | TRYPTOPHAN | 0,333 | 0,8350 | 1,03 | 0,1059 | 0,78 | 0,4610 |
| NS | URACIL | 0,310 | 0,3526 | 0,91 | 0,5290 | 1,07 | 0,0922 |
| NS | URACIL 5-CARBOXYLATE | 0,179 | 0,1548 | 0,87 | 0,1661 | 1,14 | 0,2190 |
| NS | UREIDOPROPIONATE | 0,395 | 0,8950 | 1,03 | 0,2000 | 1,33 | 0,3473 |
| NS | Val-Phe-OH | 0,267 | 0,5432 | 1,12 | 0,0715 | 1,40 | 0,7816 |
| NS | XANTHOSINE | 0,085 | 0,0281 | 0,65 | 0,1348 | 0,75 | 0,2846 |

**Supp Data 2**

Use of the ChemRICH database (Barupal and Fiehn, 2017) allows the easy grouping of significantly altered metabolites depending on their chemical similarity and the resistance of the strain (Figure below). It is therefore observed that the resistant strain has an underexpression (red circles) of cholic acids, dipeptides and disaccharides and an overexpression (blue circles) of amino-acids and fatty acids (O=FA) in the feces, compared to the standard rats.


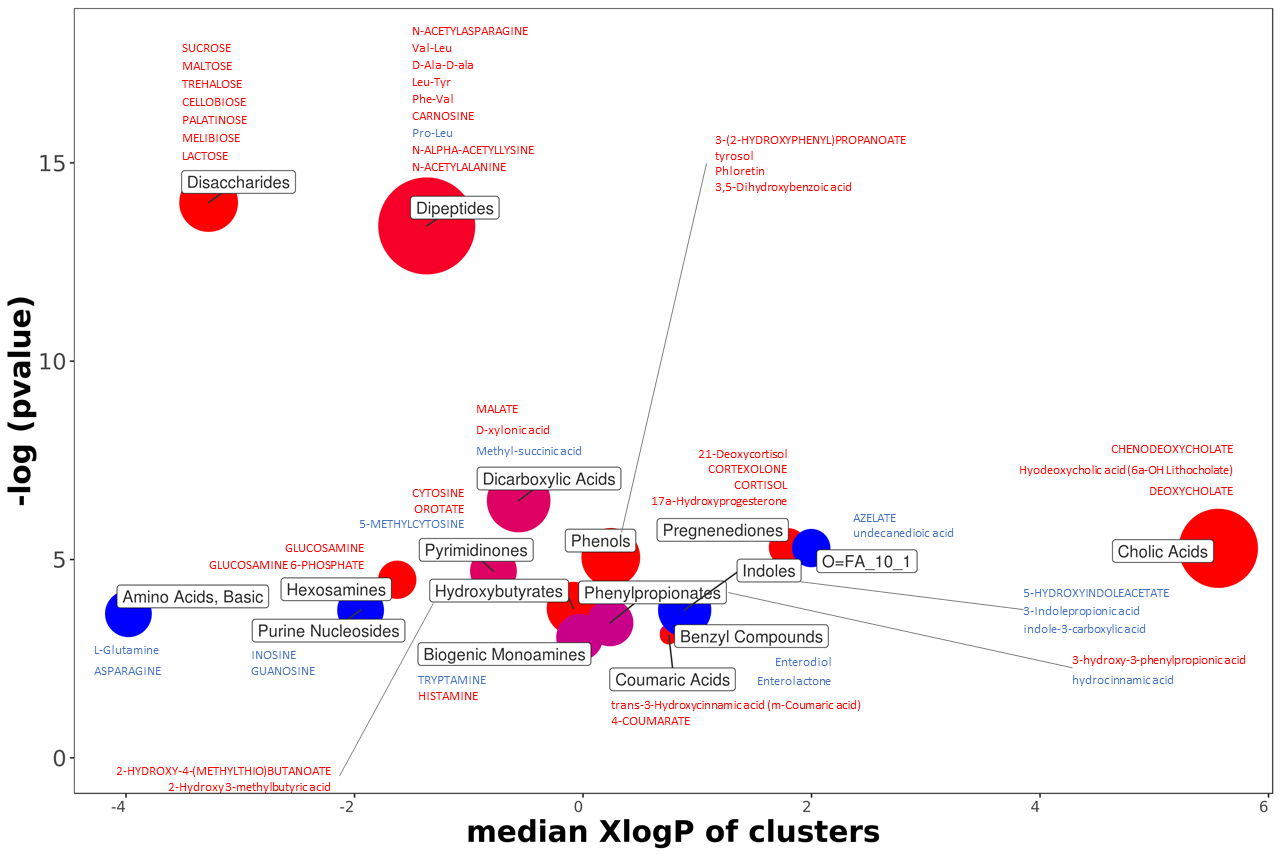


ChemRICH diagram (Barupal and Fiehn 2017) according to the strain, including the detail of compounds in each node. Each disc reflects a significantly altered family of metabolites. These groups are developed from chemical similarities highlighted by a hierarchical Tanimoto map (not shown) accessible in the ChemRICH program. Enrichment p-values are given by the Kolmogorov–Smirnov test. Disc sizes varies with the total number of metabolites. Blue or red discs present groups of overexpressed or underexpressed metabolites in Res compared to Std rats, respectively. Purple color represents both increased and decreased metabolites. For example, there are less disaccharides in the feces of the Resistant rats.

**Supp Data 3**

5HIAA/5-HT ratio in the ceca metabolome in a strain of rats resistant to decompression sickness.


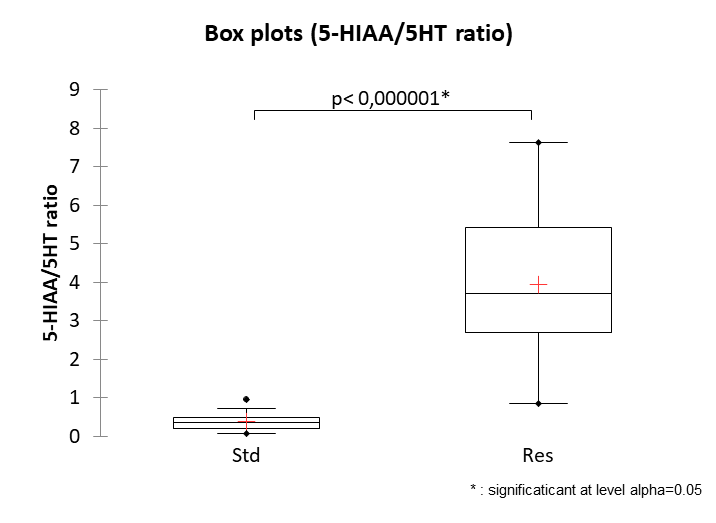

Supplement: Supplementary file 1 — Supplementary Information. [file 41598_2021_87952_MOESM1_ESM.docx]
